# Labelled dataset for Ultra-Low Temperature Freezer to aid dynamic modelling & fault detection and diagnostics

**DOI:** 10.1038/s41597-023-02808-6

**Published:** 2023-12-09

**Authors:** Tao Huang, Silas Nøstvik, Peder Bacher, Jonas Kjær Jensen, Wiebke Brix Markussen, Jan Kloppenborg Møller

**Affiliations:** 1 Section for Dynamical Systems, DTU Compute, Asmussens Allé, Building 303B, Kgs. Lyngby, 2800 Denmark; 2 Section of Thermal Energy, DTU Construct, Koppels Allé, Building 403, Kgs. Lyngby, 2800 Denmark; 3 Danish Technological Institute, Gregersensvej 1, 2630 Taastrup, Denmark

**Keywords:** Thermoelectric devices and materials, Mechanical engineering

## Abstract

Ultra-low temperature (ULT) freezers are used to store perishable biological contents and are among the most energy-intensive equipment in laboratory buildings, biobanks, and similar settings. To ensure reliable and efficient operation, it is essential to implement data-driven fault detection and diagnostic algorithms, along with energy optimization techniques. This study presents labelled and long-term ULT-freezer performance dataset, the first of its kind, derived from 53 ULT freezers featuring two different control strategies. The dataset comprises high-resolution historical operation data spanning up to 10 years. More than 10 attributes are recorded from the freezing chamber and critical locations in the refrigeration systems. The dataset is labelled with regular events, such as door openings, as well as fault events obtained from 46 service reports. A scalable data pipeline, consisting of extraction, transformation, and loading processes, is developed to convert the raw data into a format ready for analysis. The dataset can be utilized to support the development of data-driven models and algorithms that advance the intelligent digital operation of ULT freezers.

## Background & Summary

Ultra-low temperature (ULT) freezers play an essential role in the pharmaceutical business, research organizations, and food industries for preserving perishable biological materials. Their significance has been further emphasized during the Covid-19 pandemic, where they have been used for storing vaccines^1^. ULT freezers operate at temperatures ranging from −40 to −86 °C. This extreme temperature places them among the most energy-intensive equipment in hospitals, biobanks, and laboratory buildings^2,3^. A typical ULT freezer can consume up to 20 kWh per day^4^, which is nearly three times the daily power consumption of an average Danish household^5^. Given the high energy consumption, there is significant potential for improving their energy performance and optimising their operational strategies. In addition, the detection and diagnostics of faults or malfunctions in ULT freezers are also crucial to ensure the integrity and quality of stored samples.

With the fast development of digital technologies, data-driven approaches have gained increasing attention in the realm of smart surveillance and operation optimization of energy systems, particularly in domains including buildings^6–8^, urban heating networks^9,10^, and manufacturing processes^11,12^. These data-driven approaches have proven effective in safeguarding energy systems against failures through automated fault detection and diagnostic (FDD) and enhancing their working efficiency through energy flexible operations with model predictive control (MPC). Considering the increasing focus on green transitions in society, it is also imperative to incorporate data-driven techniques in ULT freezers to ensure their controllable, flexible, and reliable operations. Such integration is expected to yield not only economic benefits through reduced energy costs but also substantial social benefits through curbing carbon emissions and mitigating environmental pollution from refrigerant leakage. Therefore, the provision of a comprehensive dataset becomes crucial in facilitating these advancements.

Unlike large-scale energy systems, ULT freezers are more vulnerable to external disturbances from immediate thermal environment and human activities, resulting in continuous changes in its system dynamic states over time^13^. Consequently, short-term operational data would not be able to uncover their holistic dynamic features. Utilizing long-term data, which contains a higher level of information on practical operational features, is more suitable for data-driven models or algorithms to learn their actual dynamic characteristics under different operational scenarios. This is essential for maximising the practical applicability and sustaining the long-term performance of these data-driven approaches. However, no open-access dataset for the long-term performance of ULT freezers currently exists. This has hindered the development of data-driven models and algorithms that can support smart digital operations of ULT freezers.

Furthermore, in the context of ULT freezers, FDD can frequently hold higher significance than energy-saving, given the potentially high monetary value associated with the stored materials. However, the development of (semi-)supervised FDD algorithms typically necessitates labelled data, which provides verified ground truth information on the presence and absence of faults. The labelled data is pivotal as it facilitates training the data-driven approaches with real-world examples, enabling them to predict equipment failures or malfunctions based on specific patterns or anomalies in the time series data. In addition, the manual simulation of additional faulty scenarios that resemble actual fault events can be undertaken, thereby enriching the dataset. All these aspects can strongly contribute to the development of the FDD algorithms and proactive maintenance strategies of the ULT freezers. This augmentation, consequently, ensures the timely identification and resolution of potential issues prior to escalation, secures uninterrupted operations, and minimises the ramifications of costly downtime and potential loss of invaluable samples. Unfortunately, there is currently no labelled dataset available for ULT freezers, similar to the lack of long-term performance data. This significant gap hampers the development of reliable FDD algorithms for ULT freezers.

To address the aforementioned data challenges, this paper presents a dataset derived from 53 ULT freezers utilised in regular daily operations in Danish National Biobank. To the authors’ best knowledge, the presented dataset is the first of its kind focusing on the long-term performance of ULT freezers as well as the first labelled dataset for ULT freezers. The distinguishing element of this dataset lies in the following key features:

- Encompassing high-resolution historical operational data spanning up to 10 years;
- Including data collected from ULT freezers equipped with conventional ON/OFF and variable speed control systems;
- Containing the measurements from not only freezing chambers but also multiple critical locations in the cascade refrigeration systems;
- Labelled with regular events, such as door opening/closing, alongside fault events that derived from service reports describing the repair history of the freezers.

The dataset aims to fill the gap of accessible labelled datasets for ULT freezers and can offer various potential applications, such as:

- Promoting the understanding of long-term performance variations and gaining insight into the dynamic behaviours;
- Allowing for a detailed exploration of the relationships and synergies between different components in the refrigeration systems and the freezing chambers;
- Serving as a benchmark for evaluating simulation tools for ULT freezers;
- Allowing for recognizing pattern changes during different events at different locations in the refrigeration systems and promoting the understanding of the evolving patterns leading from a soft fault to a hard fault and, potentially, a failure;
- Guiding the fault diagnosis algorithms developments as well as the design of resembling faulty scenarios for further analysis;
- Facilitating the development and validation of dynamic models employed for predictive control and maintenance of ULT freezers;
- Supporting the development of reliable and transferable FDD and MPC algorithms and contributing to the enhancement of ULT freezer reliability and energy efficiency.

## Methods

### ULT Freezers

**Fig. 1:**
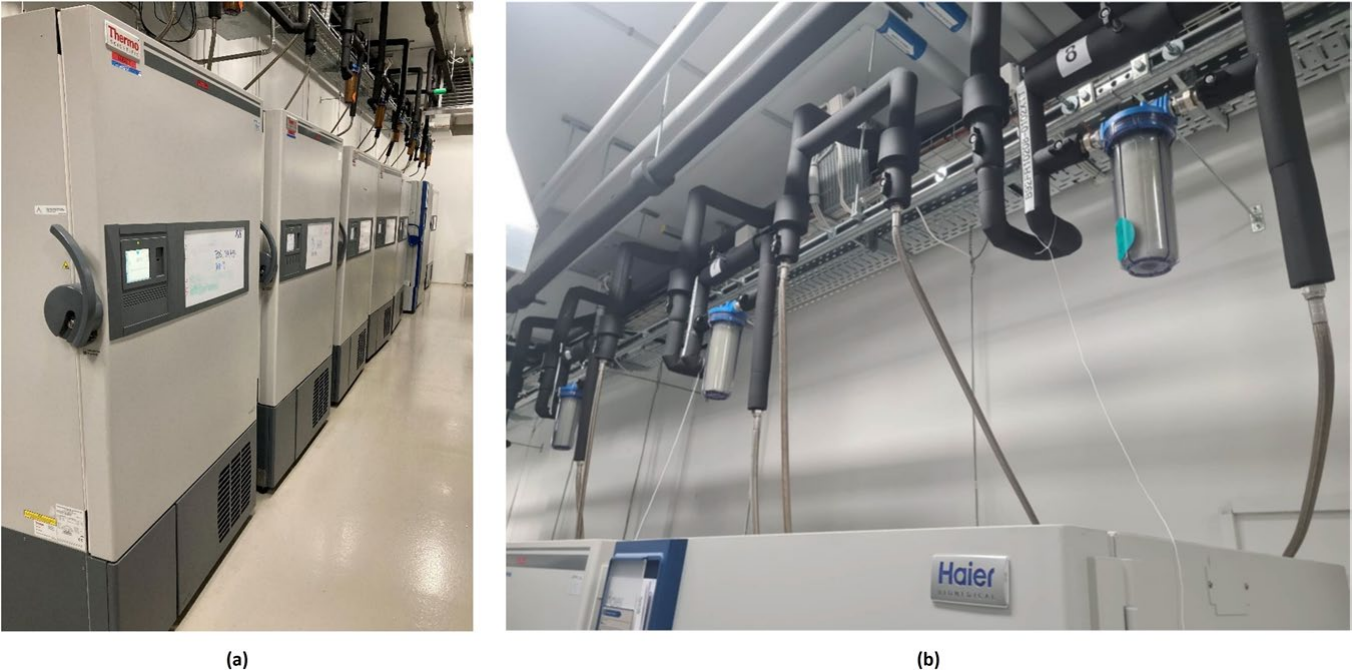
(**a**) The room accommodating 53 ULT freezers. (**b**) The insulated chilled water pipes and multi-particle filters.

The data were collected from 53 ULT freezers operated by the Danish National Biobank. All ULT freezers are located in a room where the air temperature is conditioned using fan coils, see Fig. 1a. Among the 53 ULT freezers, 38 freezers were manufactured by Thermo Fisher Scientific with model version Revco UxF70086V (referred to as Revco freezers), while remaining 15 freezers were manufactured by Haier with model version DW-86L959W (referred to as Haier freezers). Both manufacturers are well-regarded for producing ULT freezers, and their products are widely sold worldwide. The Revco freezers have been in operation since 2012, while the Haier freezers are relatively newer and have been in operation since 2021–2022. All freezers are identified by a six-digit ID number, e.g. 806016.

**Fig. 2:**
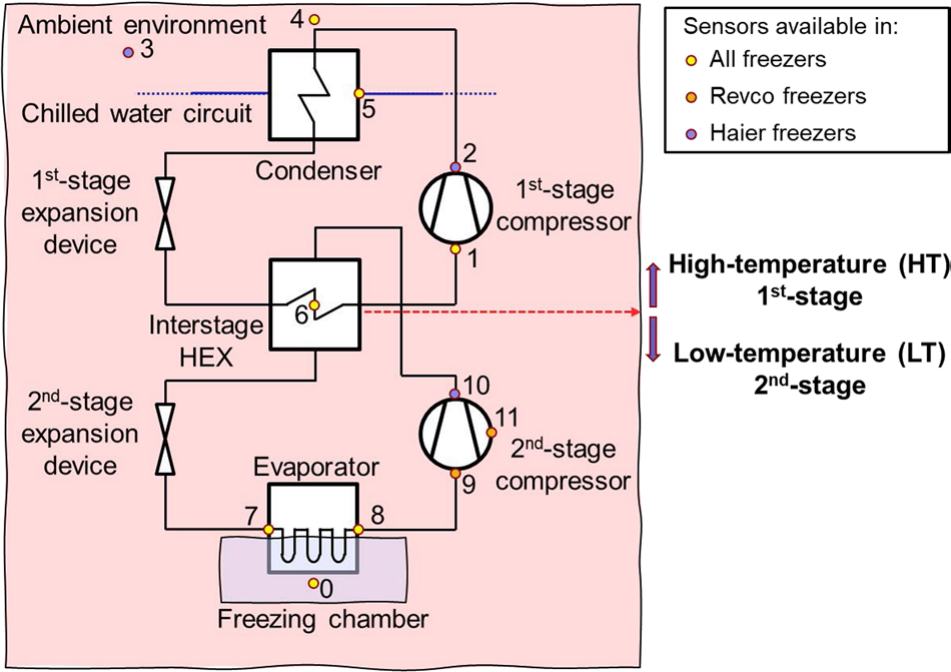
Schematic of the working principle of 2CRS and sensor locations in two types of ULT freezers. Refer to Table 2 for respective sensor indices.

The two types of freezers have comparable interior chamber dimensions (W × D × H): 1016 × 716 × 1310 mm for Haier freezers and 1016 × 686 × 1300 mm for Revco freezers, which are typical sizes used in the relevant professional settings. The freezing chamber has 4 shelves, each with its own thermal insulated door, in addition to the external door. Both types of ULT freezers are equipped with a Two-Stage-Cascade Refrigeration System (2CRS). The working principle of the 2CRS is illustrated in Fig. 2. The 2CRS comprises two separate refrigeration cycles: a high-temperature stage named 1^st^ stage and a low-temperature stage, named 2^nd^ stage. Each stage includes a compressor, a condenser, an evaporator and an expansion device. These two stages are connected through an inter-stage heat exchanger (HEX), which acts as the evaporator in the 1^st^ stage and the condenser in the 2^nd^ stage. In both ULT freezers, hermetic reciprocating compressors are used. The Revco freezers have fixed-speed compressors regulated by thermostatic ON/OFF controllers, while the Haier freezers are equipped with variable-speed compressors. The working fluids used for the two ULT freezers are different. Please refer to Table 1 for detailed information regarding the compressor models and refrigerants used by two types of ULT freezers.

**Table 1:** Information on the compressors and refrigerant of the ULT 4Freezers.

Freezer model	Quantity	1^st^ stage compressor	2^nd^ stage compressor	Refrigerant (1^st^/2^nd^*)
Revco UxF70086V	38	Copeland RFT42C1E-PFZ-382	EmbracoNT2212GKV	R404A/R508B + R290
DW-86L959W	15	Embraco VNEU217U	Embraco VNEU217U	

* The 2^nd^ stage uses a blend of two refrigerants. For Revco freezers, the blend comprises a mixture in the ratio of 7.1% R290 and 92.9% R508B. For Haier freezers, the blend consists of a ratio of 8.2% R290 and 91.8% R508B.

All ULT freezers are cooled by water, meaning the condensers exchange heat with a separate chilled water circuit. The chilled water pipes are connected to each freezer in parallel, see Fig. 1b. Prior to December 2019, the chilled water temperature was maintained at approx. 10 °C, afterwards the water temperature is maintained at 20 °C throughout the year. To reduce the calcium content and air bubbles, multi-particle filters are installed on the chilled water branch pipes for each freezer, see Fig. 1b. Furthermore, a chemical controller is installed on the main water circuit to regulate the pH values, thereby mitigating the presence of metal particle impurities in the pipes. The multi-particle filters are replaced four times per year to prevent fouling problems and preserve cooling performance. The water-based cooling system also eliminates the issue of air intake blockage by dirt, a common occurrence in air-cooled systems.

### Data acquisition

#### Raw data collection and description

Both the Revco and Haier freezers are integrated with multiple temperature sensors located in various positions by the manufactures, see Fig. 2. In addition, a logging system is integrated into both freezers, allowing the recorded measurements to be stored for a minimum of 15 years. Table 2 presents the attributes measured by the two types of ULT freezers. Apart from the chamber temperature, which is measured by a Resistance Temperature Detector (RTD), all other temperatures are measured using standard thermocouples. These temperature measurements are taken at critical positions in the refrigeration loops, which facilitates performance monitoring and fault identification. However, certain measuring positions appear in only one type of freezer, such as 2^nd^ stage compressor sump temperature for Revco freezers and the compressor discharge temperatures in Haier freezers.

**Table 2:** Recorded attributes from two types of ULT freezers.

Attributes	Revco freezers	Haier freezers	Unit
Datetime	✓	✓	Y-m-d H:M:S
0: Chamber temperature	✓	✓	°C
1: 1^st^ stage compressor suction temperature	✓	✓	°C
2: 1^st^ stage compressor discharge temperature		✓	°C
3: Ambient air temperature		✓	°C
4: Condenser air inlet temperature	✓	✓	°C
5: Chiller water inlet temperature	✓		°C
6: Interstage heat exchanger temperature	✓	✓	°C
7: Evaporator inlet temperature	✓	✓	°C
8: Evaporator outlet temperature	✓	✓	°C
9: 2^nd^ stage compressor suction temperature	✓		°C
10: 2^nd^ stage compressor discharge temperature		✓	°C
11: 2^nd^ stage compressor sump temperature	✓		°C
Setpoint temperature	✓	✓	°C
Main voltage	✓	✓	*V*
Freezer state signal	✓		
Event logs	✓	✓	

A notable difference in the temperature measurements obtained from the refrigeration systems is that the values recorded by the Revco freezers are floating-point numbers with one decimal precision, while the values recorded by the Haier freezers are integers. Furthermore, the sampling rate for measurements in Revco freezers is 1 min, whereas it is 6 min for Haier freezers.

The raw data collection process was quite time-consuming due to the manual extraction. The data had to be downloaded individually from each freezer using a USB drive. Extracting the data from the 15 Haier freezers, being the newer models, took approximately 1 h. The extracted raw data was in .db format, stored as an SQLite database consisting of multiple tables containing temperature measurements and event logs.

However, the raw data extraction process from the Revco freezers presented some limitations. Due to the restrictions imposed by the freezers, the data for nearly a decade could only be exported in 6-month intervals at a time. Consequently, extracting the data from each 6-month data period of a single freezer took approximately 8 min. Moreover, the event log data had to be downloaded separately from the temperature data, further prolonging the data collection time. The exported raw data is in .dat format as separate files.

A rather useful “State” signal is recorded by the Revco freezers. The “State” signal reflects what components are activated by the control system, see (1). It is represented as an integer but should be interpreted in a binary manner. In the binary code, 0 signifies a component that is not energised, while 1 represents an energised component. The state signal is a sum value ranging from bit zero (*b*0) to bit six (*b*6), as represented in (1). For example, the most common and meaningful state signals are 1 (0000001) and 3 (0000011), which indicates that only the 1^st^ stage compressor is activated and compressors on both stages are activated, respectively. Thus, the transition between the state signal 0 to 1 indicates the initiation of a duty cycle and vice versa.

1 $$\mathop{\underbrace{b6}}\limits_{\begin{array}{c}{\rm{Backup}}\;{\rm{system}}\\ {\rm{tank}}\;{\rm{status}}\end{array}}\;\mathop{\underbrace{b5}}\limits_{\begin{array}{c}{\rm{Backup}}\;{\rm{system}}\\ {\rm{injecting}}\;{\rm{status}}\end{array}}\;\mathop{\underbrace{b4}}\limits_{\begin{array}{c}{\rm{Door}}\;{\rm{status}}\end{array}}\;\mathop{\underbrace{b3\;b2}}\limits_{\begin{array}{c}{\rm{Incoming}}\\ {\rm{voltage}}\;{\rm{status}}\end{array}}\;\mathop{\underbrace{b1}}\limits_{\begin{array}{c}{\rm{2nd}}\;{\rm{stage}}\\ {\rm{compressor}}\end{array}}\;\mathop{\underbrace{b0}}\limits_{\begin{array}{c}{\rm{1st}}\;{\rm{stage}}\\ {\rm{compressor}}\end{array}}$$

In addition to the numeric data, certain events that occurred at the freezers are also logged. The event logs obtained from the Revco freezers consist of three columns describing the event in detail, including “Type”, “Event”, and “Description”. The “Type” column indexes a unique event class (e.g. [3]), the “Event” column displays the name of the event class (e.g. “Shutdown”), and the “Description” column provides further information about the events. The column “Description” is merged with the “Event” column during data processing for better readability. The event logs from the Haier freezers contain only one column indicating the event name. In the raw data, there are 59 event classes for the Revco freezers and 7 event classes for the Haier freezers. These event classes can be accessed in Data Records.

#### Faulty events collection from the service reports

In addition to the data recorded by the freezers themselves, a total of 83 service reports were collected from a third-party service company responsible for conducting repairs and scheduling service inspections. These service reports, written in Danish, pertain exclusively to the Revco freezers as the Haier freezers are still under warranties and had not required any services. The service reports are in .pdf format and include 37 reports for regular inspection and 46 reports for fault repairs. The oldest service report for repair dates back to 2014-07-29, while the most recent one is from 2023-04-12.

**Fig. 3:**
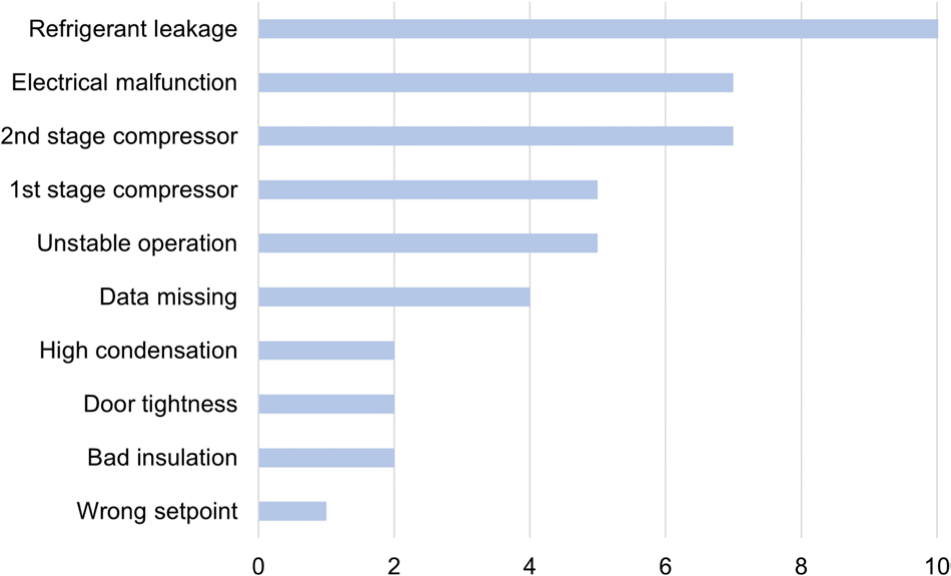
Count of categories of faults summarised from the service reports.

The fault repair reports provide descriptions of the symptoms, malfunctional components, and the corresponding solutions. In addition, the replaced parts are also listed. This information proves valuable as it enables further labelling the data with specific fault events. However, the fault types are not well categorized in the reports by the service company. Upon careful examination of the service reports, seven types of faults are summarized, see Fig. 3. Among these, the most frequently occurring fault is the Refrigerant Leakage, succeeded by electrical malfunctions like fuse blow and wiring issues, along with compressor malfunctions in both stages. Note that, the data is missing for four fault events. Thus a total of 42 events are labelled in 24 Revco freezers. Detailed information on all fault events is summarised in a spreadsheet and can be found along with Data Records.

### Data ETL pipelines

The data is processed through a data pipeline, which is a digital process through which raw data from different sources is processed and transformed to make it ready for analysis. The present study specifically applied an ETL (Extracted-Transformed-Loaded) pipeline, which is a specific subcategory of data pipelines that follows a sequential order of operations. First, the raw data is *Extracted* from various sources. Then, the extracted data is *Transformed* into an analysis-friendly format. Finally, the transformed data is *Loaded* into its intended destination, like a database, data warehouse, or data lake^14^.

The ETL pipeline for the ULT freezer data is developed using separate Python utility functions. The Extract.py, which contains a RevcoExtracter and a HaierExtracter, is responsible for extracting the freezer-brand-related data from different formats and directories and loading it into a data frame. In addition, it identifies all observed event classes and can locate missing and corrupted files. Due to the limitations of the selected ULT freezers, the data is fed through the data pipeline in batches.

The Transform.py file performs the necessary transformations on the extracted data to a format suitable for analysis. Similarly, it contains a RevcoTransformer and a HaierTransformer. Due to differences in the raw data structure, distinct methods are employed to perform the transformations for the two types of freezers. The transforming process includes operations such as casting data types, removing duplicates, renaming columns, calibrating the timestamps, and filtering out events with no interest. The original column names of the raw datasets and the renamed column names from two types of ULT freezers are tabulated in Table 3. Timestamp calibration was performed to rectify time deviations between the set time and the actual time in specific freezers due to inaccuracies in the system time settings. Subsequently, the temperature data are labelled with regular events from event logs and fault events from the service reports. Note that events are logged at the exact time they occured, the exact timestamps in seconds for event and fault labels may not align with those for temperature timestamps, as temperature recordings are captured on a per-minute basis. Hence, the merge procedure searches for the closest temperature timestamp for each event and fault label. In addition, the outliers present in the raw dataset were intentionally kept. This decision was made because the freezing room is inspected regularly, and any irregular readings in the temperature measurements are likely attributed to external disturbances such as human activities or technical malfunctions. Thus, preserving these data allows for the potential retention of underlying information.

**Table 3:** Column names of raw data and processed data.

Description	Original		Renamed
	Revco	Haier	
0: Chamber temperature	RTD	RTD	RTD
1: 1^st^ stage compressor suction temperature	TC1	H_IN	1st Suc.
2: 1^st^ stage compressor discharge temperature		H_OUT	1st Disc.
3: Ambient air temperature		AMBIENT	Ambient
4: Condenser air inlet temperature	TC2	CONDENSER	Cond. Air In
5: Chiller water inlet temperature	TC7		Chil. Water In
6: Interstage heat exchanger temperature	TC10	HOT_EXCHANGER	H.E.
7: Evaporator inlet temperature	TC3	EVAPORATOR_IN	Evap. In
8: Evaporator outlet temperature	TC4	EVAPORATOR_OUT	Evap. Out
9: 2^nd^ stage compressor suction temperature	TC6		2nd Suc.
10: 2^nd^ stage compressor discharge temperature		L_OUT	2nd Disc.
11: 2^nd^ stage compressor sump temperature	TC9		2nd Sump

Finally, the Load.py file is responsible for storing the processed data in a retrievable format. In this study, the output data format is .parquet^15^, a binary format compatible with most of the popular programming languages for data analysis.

## Data Records

**Fig. 4:**
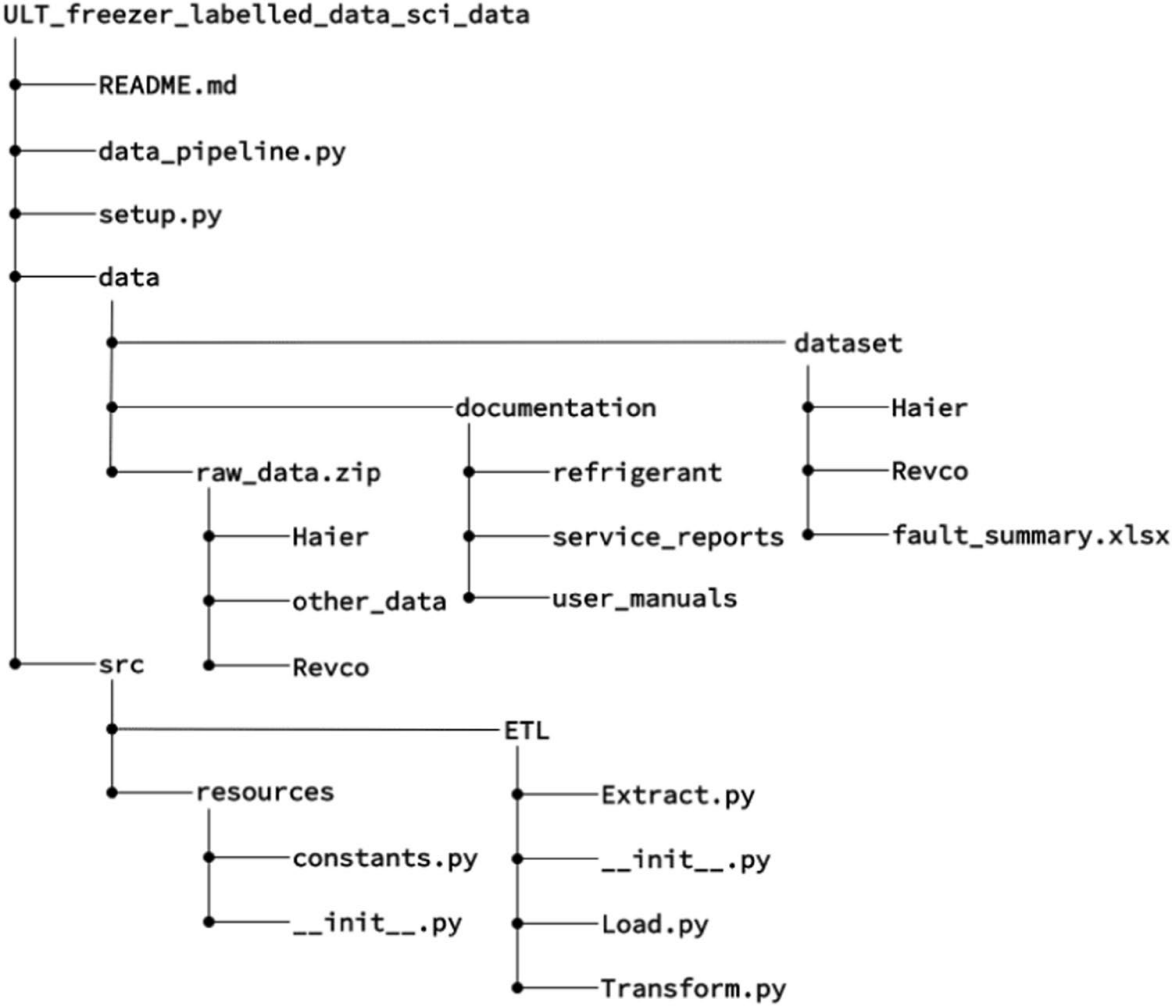
Data repository structure.

The data processed through the ETL pipelines along with the Python scripts for ETL algorithm are stored in *figshare*^16^ and also in an open-access GitLab repository^17^. The overall structure of the repository is presented in Fig. 4. The repository contains two main folders: data and src. A Python script named data_pipeline.py can also be found, which invokes three ETL utility functions, meaning the complete ETL pipeline can be easily run by executing data_pipeline.py. Additionally, the repository includes a setup.py file and a README.md file for reference and setting up required environments, respectively.

In the data folder, the archive file raw_data.zip contains all the necessary raw data files, including the raw data for both types of ULT freezers. The processed data is available in the dataset folder, which includes two subfolders named Haier and Revco. Each subfolder contains the processed data in the format of.parquet. The.parquet files are named using the format [freezer index]_[events/temp], where 'events' or 'temp' represent the event logging and merged labelled data, respectively. The event classes are also summarised in respective folders named distinct_event_classes.txt. Additionally, the ‘dataset’ folder includes the file fault_summary.xlsx, which summarises fault events, including associated dates, fault conditions, and categories. This information is extracted from the service reports by the authors. Within the data folder, the documentation folder contains a subfolder named refrigerants, which includes Pressure-Enthalpy diagrams for three refrigerants used by the ULT freezers for potential thermodynamic analysis. It also includes a subfolder named service_reports, containing all the service reports in .pdf format from the service company. Lastly, there is a subfolder named user_manuals, which contains the manuals (brochures) for the two types of ULT freezers provided by the manufacturers.

Within the src folder, two subfolders named ETL and resources can be found. The ETL folder contains the three ETL pipeline functions, which are responsible for extracting, transforming, and loading the data. The resources folder includes a file named constants.py that contains the parameters used in the ETL pipeline.

## Technical Validation

**Fig. 5:**
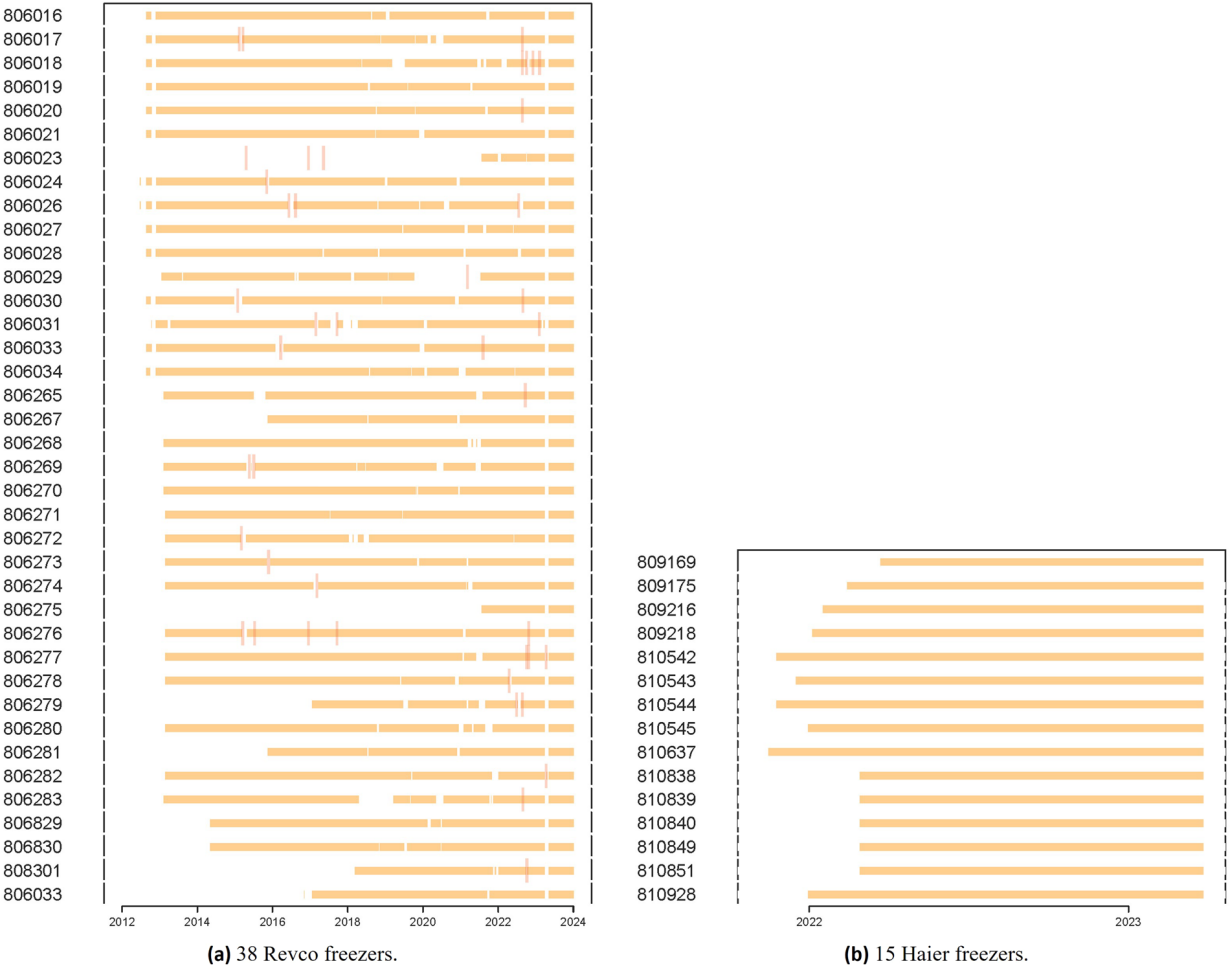
Availability of the data through ETL pipelines for 53 freezers. Gaps in the lines represents a period where data is missing. The fault events from the service reports are marked with a vertical bar.

Figure 5 displays the availability of the time series data of all ULT freezers based on chamber temperature (RTD) measurements. A break is added when there is a missing data recording. The Haier freezers, which have not undergone any services, do not have any breaks in their data. Additionally, fault events extracted from the service reports are depicted with a vertical bar in Fig. 5a to demonstrate their temporal occurrences. Figure 5a shows that some shutdowns align directly with the faults, while others can be resolved without disrupting operations. Therefore, data breaks occurring without associated faults are presumably linked to other factors, such as maintenance and standby periods when ULT freezers are not in use. Unfortunately, maintenance activities like defrosting are not documented. Eventually, the datasets for the Revco freezers vary in length, with the longest dataset (No. 806020) containing 5,489,043 observations per attribute and the shortest dataset (No. 806023) containing 847,575 observations per attribute. The Haier freezers have a shorter overall data length, with the longest dataset (No. 810637) containing 119,307 observations per attribute and the shortest dataset (No. 809169) containing 88,582 observations per attribute. The non-compressed raw data has a size of 16.9 GB, while the processed dataset has a size of 5.6 GB.

The collected data has been obtained from routine operations over the past several years, rather than from controlled experiments. As a result, it cannot be strictly validated against any benchmark experiments. Therefore, the technical validation for the presented dataset is conducted by assessing the physical meaning and interpretation of the time series data, specifically with respect to four scenarios: (1) Duty cycle variation, (2) regular operational patterns, (3) operational patterns during door events, (4) operational pattern during typical fault events. Representative examples from selected freezers are used to demonstrate each scenario. The qualification of the data collection systems is guaranteed by the manufacturers, and thus should not be a concern.

### Variations in the duty cycle lengths in Revco freezers

**Fig. 6:**
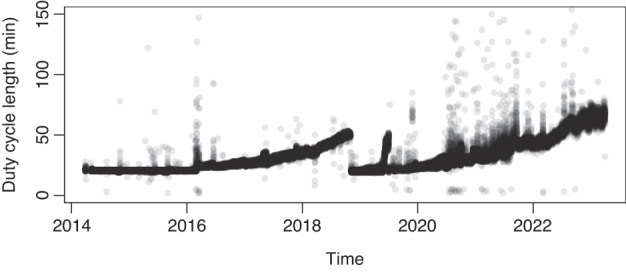
Evolution of the duty cycle length of Revco Freezer No. 806830.

Due to the ON/OFF control of the Revco freezers, the duty cycle lengths can be quantified. Figure 6 exemplifies the duty cycle lengths of Revco freezer No. 806830. The plot exhibits an overall increasing trend of duty cycle lengths, with occasional spikes indicating significantly longer duty cycles. These spikes correspond to door-opening events, as indicated by the event logs. From 2014 to 2016, the duty cycle length remains relatively stable. Subsequently, it gradually increases to approximately 50 min by 2019. At this point, a data break occurs, which aligns with what is shown in Fig. 5. When the freezer is turned on again, the duty cycle length returns to a comparable level from 2014 and then gradually increases again.

The data break period does not indicate any equipment failures, as confirmed by the service reports. Instead, it is most likely attributed to the manual defrosting activity. The ULT freezers used for storing perishable biological contents typically lack automated defrosting functions to ensure content quality. Therefore, the selected freezers require manual defrosting. Moreover, as shown in Fig. 6, door-opening events have a significant impact on the increase of duty cycle lengths. In 2021, when intensive door-opening events occur, the duty cycle length increases noticeably. This is reasonable since direct contact with warm ambient air leads to ice buildup on the interior chamber surface, thereby increasing the cooling load. Consequently, the duty cycle lengths continue to rise to meet the cooling demand and reach nearly 70 min towards the end of the time series. Overall, the observed phenomena align with the practical expectation.

### Typical regular operational patterns

**Fig. 7:**
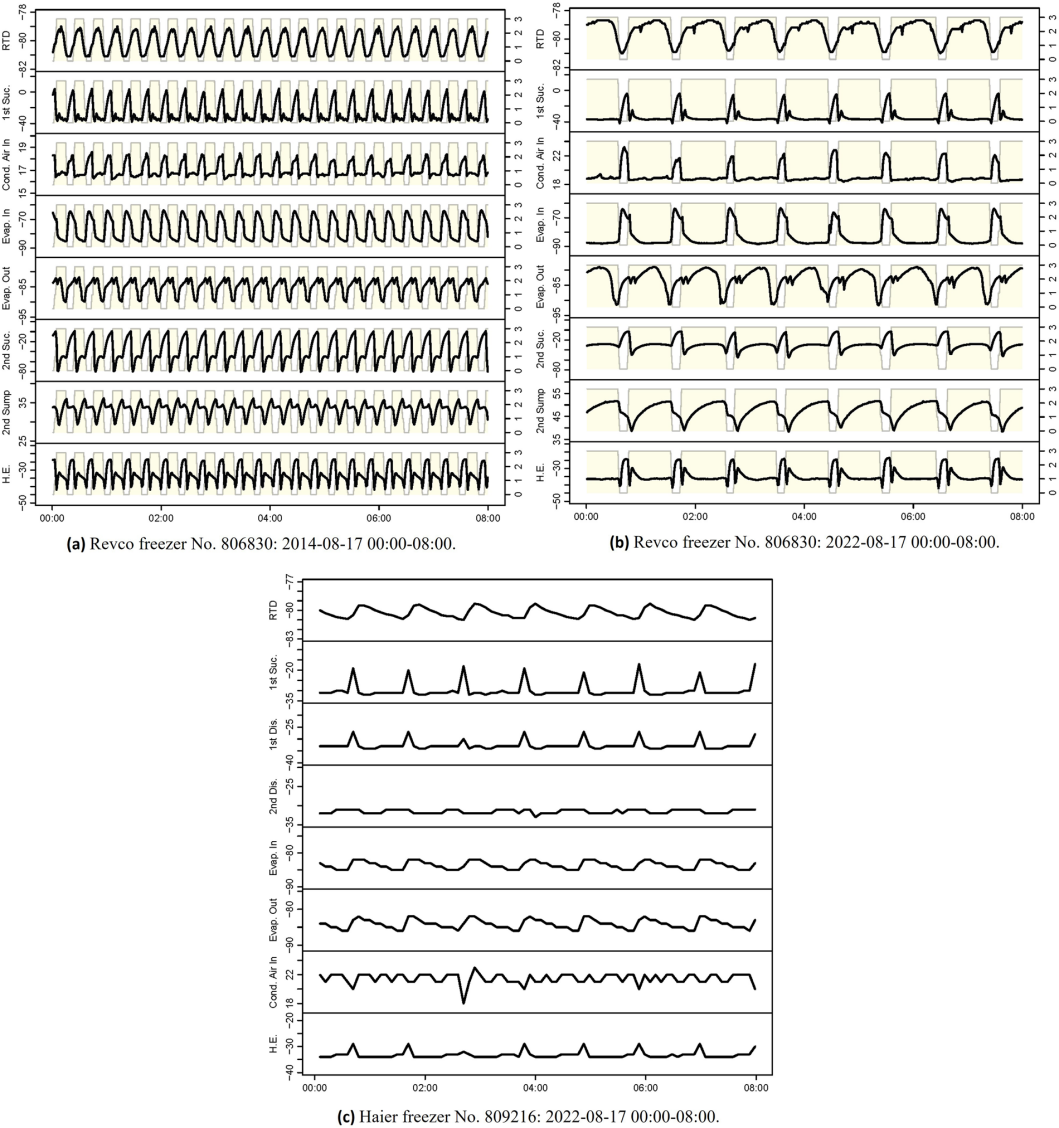
Temporal evolution of the temperatures during regular operations.

Figure 7a,b showcase the operational patterns during two 6 h periods with different duty cycle lengths for the Revco freezer No. 806830. It could be observed that the duty cycle length has a significant impact on the temperature dynamic patterns. Moreover, a notable feature is that the chamber temperature measured by RTD shows a delayed response compared to shifts of the state signals. When the state signal transitions from 0, the chamber temperature still keeps rising. The slope of the increase gradually reduces and becomes flat for a period before the pull-down phase begins. This distinct chamber temperature profile is consistent across all Revco freezers. The delayed response can be explained by the thermal inertia between the evaporator and the chamber. The mismatch between the pull-down duration and the ON state span is due to the placement of the RTD sensor. The RTD is placed in the lower part of the chamber. Experimental results reported in^18^ indicate that the temperature measurements in the lower part of ULT freezing chambers tend to be consistently higher and exhibit slower responses compared to measurements from the core regions. This discrepancy arises because the lower part of the chamber is close to the outlet of the evaporator, where most refrigerants have become gaseous, leading to a higher evaporation temperature and lower cooling intensity. Therefore, the RTD temperature can be inferred to represent a local temperature and possibly the warmest temperature in the chamber. Despite this bias, the location ensures that the entire chamber remains below the temperature setpoint, making it a signal suitable for control and fault detection purposes. Additionally, notable correlations are observed among the responses of different temperature evolution. All temperatures measured from the refrigerant loops show a noticeable increase during the OFF period when the refrigerant circulation ceases, which is expected in practice. In contrast, the 2^nd^ stage oil sump temperature experiences a reduction during the OFF period. This is due to no piston friction^19^. These phenomena show reasonable alignment with the physical understanding, supporting the validity and usefulness of the dataset.

Figure 7c depicts an 8 h regular operation period for a Haier freezer No. 809216. Despite utilizing variable-speed compressors, Haier freezers still exhibit periodic operational patterns. This observation suggests that the compressors of the Haier freezers may still undergo ON/OFF regulations, with the variable adjustments in the compressor speed occurring only during running time. The periodic patterns can also be observed in temperature measurements at other locations, indicating that the operational patterns of both types of ULT freezers tend to exhibit periodicity regardless of the compressor operational methods. Consistently, there are strong correlations between the dynamic responses of temperature measurements, which are physically meaningful.

### Operational pattern during door events

**Fig. 8:**
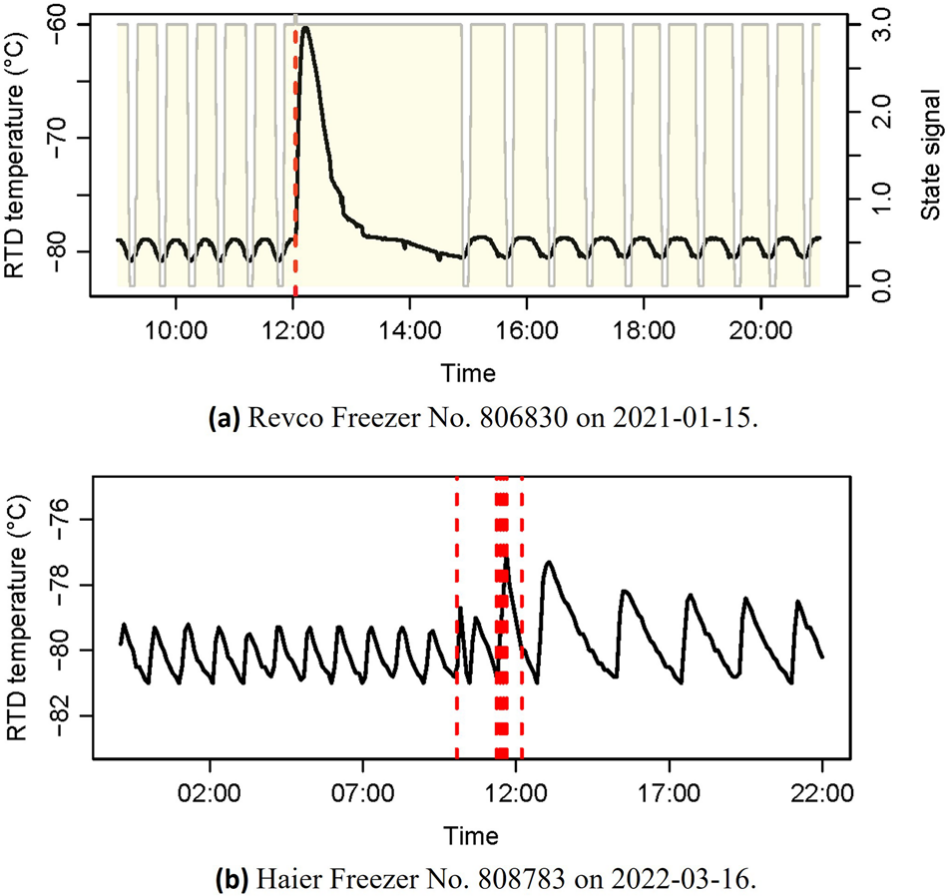
Temporal evolution of the chamber temperature during door opening events.

The door opening/closing is the most typical regular event recorded by the ULT freezers. Figure 8 shows the temporal evolution of the chamber temperature for two ULT freezers in response to door-opening events. As expected, the chamber temperature exhibits a rapid increase immediately after the door is opened. The logging system of the Revco freezers registered the time of the opening and closing activities, along with the opening duration. The logged event description indicates that the opening duration for the event shown in Fig. 8a is 54 s, which is relatively long. Consequently, the chamber temperature rises significantly to approximately −60 °C and takes nearly 1 h to reach the desired setpoint level of −80 °C.

For the Haier freezer, the door opening results in a chamber temperature increasing to approximately −77 °C, which is only a few degrees higher than the setpoint (−80 °C). Despite registering 6 consecutive door-opening activities, the chamber temperature remains relatively low. This could be attributed to the possibility of short opening duration (16 s–27 s) or the high-efficiency variable-speed compressors.

Figure 8 demonstrates the observed temperature profiles following the door-opening events align well with the expected physical behaviour, validating the usefulness of the temperature measurements.

### Operational pattern at a fault event

**Fig. 9:**
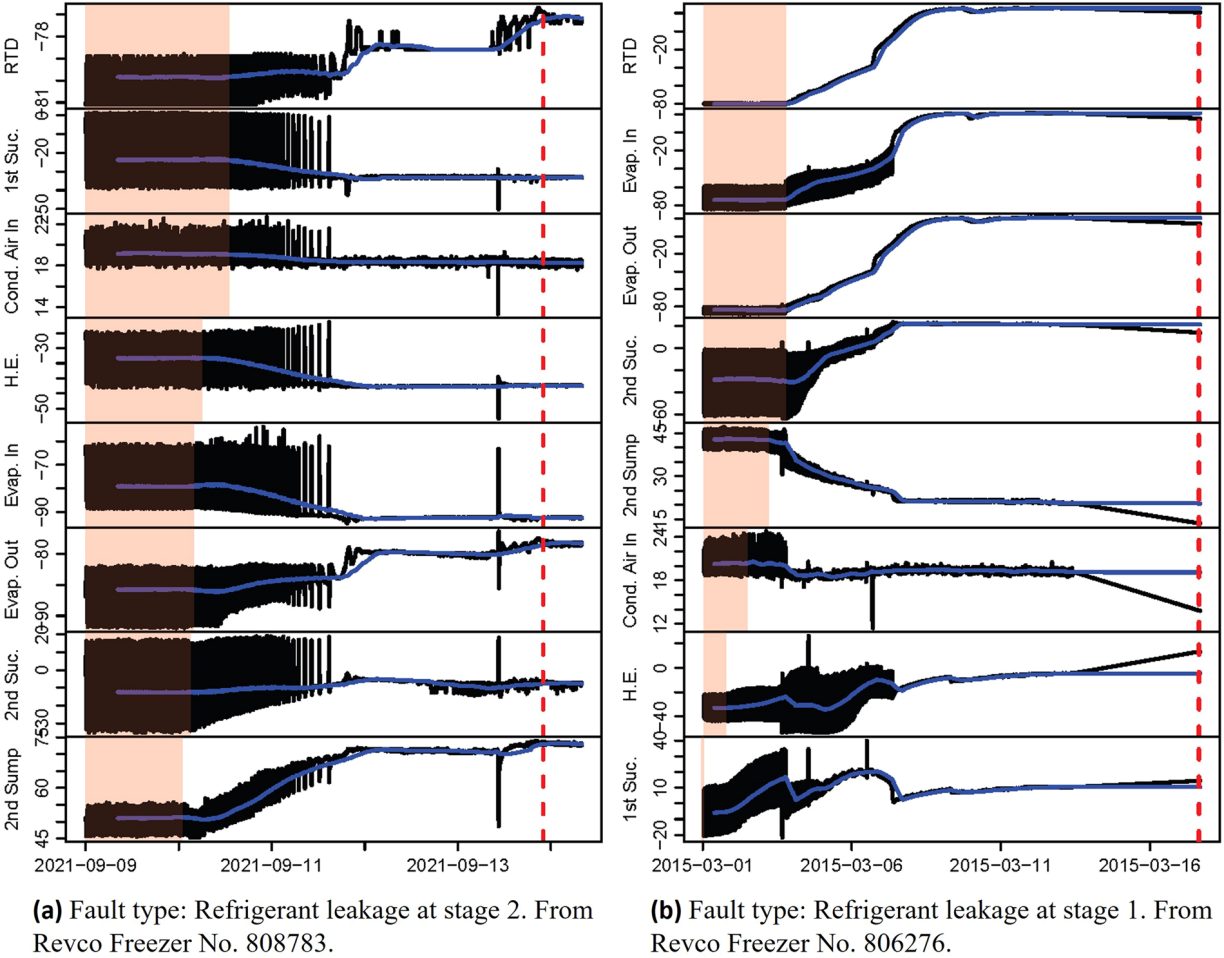
Temporal evolution of the temperatures during faulty events. The blue lines are moving average values with a sliding window width of 500 min. The shaded areas represent the regular pattern during the fault-free period.

Figure 9 illustrates the operational patterns during the most prevalent fault event: Refrigerant Leakage. To provide a better insight, we present the performance during Refrigerant Leakage happening in both stages. Note that, a limitation of the service reports is that the exact occurrence time of the faults is unknown. The fault time recorded in the service reports represents the time when the service company receives the request from the Biobank, which is likely delayed relative to the actual fault occurrence. Therefore, in the data processing procedure, the fault events are labelled at 12:00:00 on the date registered in the service reports.

Figure 9a illustrates the temporal evolution of the temperatures during refrigerant leakage at the 2^nd^ stage compressor. The vertical dashed line is the time reported in the service report for this fault event. However, the irregular temperature patterns indicate that the fault had been occurring for several days prior to the reported time. Therefore, this time should not be used to represent the actual fault occurrence time. Instead, earlier data should be examined to identify when the temperature patterns start showing irregular changes.

To highlight the regular operation period before the occurrence of the fault, we have shaded a specific area in each plot. The plots are deliberately ordered based on when the 500-min moving average values start to change. This metric is selected subjectively for demonstrative purposes. More sensitive approaches can be used for more precise identification of the occurrence time. Figure 9a shows that all temperatures during refrigerant leakage at the 2^nd^ stage compressor display noticeable changes in their patterns, with the 2^nd^ stage sump temperature being the first to exhibit irregular behaviour, followed by other temperatures, and the RTD chamber temperature being the last. Since the fault occurred in the 2^nd^ stage, it is physically plausible that all temperatures measured from the 2^nd^ stage show faster responses than those from the 1^st^ stage.

Figure 9b presents the temperature patterns during refrigerant leakage at the 1^st^ stage compressor caused by the valve malfunction. As the fault occurred in the 1^st^ stage, the temperatures related to the 1^st^ stage show faster responses. Consistently, the RTD temperature appears to be the last to respond to the faults. This delay can be attributed to the thermal inertia of the chamber, implying that chamber temperature may be a suboptimal indicator for fault occurrence. The observed physically plausible phenomena further support the validity of the data.

## Usage Notes

The data has undergone necessary transformation, aggregation, and labelled treatments, making it suitable for various applications in understanding the dynamic behaviours and advancing the digital operations of the ULT freezers, such as dynamic model establishment and FDD and MPC algorithms developments. The processed data is stored in .parquet format, which can be read, analyzed and further processed using mainstream programming languages, like Python and R.

The provided code for the ETL pipeline is written using principle of object-oriented programming and is thoroughly documented with docstrings to facilitate understanding for users. The overall ETL pipeline structure is written in an object-oriented programming manner, allowing for easy modification and scalability to other data sources with similar raw data structures. Furthermore, we plan to keep expanding the dataset with more data and service reports in the future to further enhance its diversity and utility.

It is important to note that the fault labels in the dataset are based on the registered time of service requests, which may not accurately represent the actual fault occurrence time. Users should exercise caution in this aspect. In Fig. 9, the authors have used the moving average to estimate the likely occurrence time of the fault. However, this metric may not be sufficiently robust and sensitive. More advanced approaches, such as Motif Discovery^20^ and Hidden Markov models^21^, can be employed for more precise pattern recognition, particularly when changes are subtle.

In Haier freezers, it should be noted that the temperatures measured at the refrigeration system are stored as integers. This representation of temperatures can result in the loss of information on the temperature dynamics, as depicted in Fig. 7c. Therefore, analysts should be aware of this when utilizing this data for analysis. To mitigate this issue, techniques such as smoothing or interpolation can be considered to recover some of the lost dynamics.

The identification of fault events in this study involved a manual summarization of service reports, a process that proved time-consuming and burdensome, particularly when dealing with a substantial number of service reports in the future. To enhance efficiency, automating the event summary process through the application of character identification techniques, such as Optical Character Recognition (OCR), is advisable.

The data has great potential for modelling the heat dynamics of the ULT freezer due to the presence of diverse dynamic operational behaviours. This is crucial for ensuring the model identifiability as the short-term data may have a low level of information on the systems^22^. When utilizing the data for dynamic modelling, it is suggested to use smoothed data, particularly for Revco freezers with a sampling resolution of 1 min. This can improve the stability of the model identification, as the unsmoothed data may contain larger random noise or short-frequency fluctuations. In addition, down-sampling the data from Revco freezers can also be considered.

## Data Availability

The Python code for ETL pipelines is available on the open-access GitLab at https://lab.compute.dtu.dk/taohu/ult-freezers-labelled-dataset-sci-data.git.

## References

[1] Center for Disease Control and Prevention (CDC). Pfizer-biontech covid-19 vaccine storage and beyond-use date tracking labels. https://www.cdc.gov/vaccines/covid-19/info-by-product/pfizer/downloads/Pfizer-Storage-Labels.pdf (2023).

[2] Gumapas LAM, Simons G (2013). Factors affecting the performance, energy consumption, and carbon footprint for ultra low temperature freezers: case study at the national institutes of health. World Review of Science, Technology and Sustainable Development.

[3] Copenhagen University. Plug load test for ult freezers: 20-22% lower energy consumption at −70 °C compared to −80 °C. https://baeredygtighed2030.ku.dk/pdf/frysertest.pdf (2017).

[4] Farley, M., McTeir, B., Arnott, A. & Evans, A. Efficient ult freezer storage. https://www.ed.ac.uk/files/atoms/files/efficient_ult_freezer_storage.pdf (2015).

[5] Kitzing, L., Katz, J., Schrönder, S. T., Morthorst, P. E. & Andersen, F. M. The residential electricity sector in denmark: A description of current conditions. https://orbit.dtu.dk/files/121099206/The_residential_electricity_sector_in_Denmark.pdf (2016).

[6] Chen Z (2023). A review of data-driven fault detection and diagnostics for building hvac systems. Applied Energy.

[7] Zhan S, Chong A (2021). Data requirements and performance evaluation of model predictive control in buildings: A modeling perspective. Renewable and Sustainable Energy Reviews.

[8] Yang S (2021). Model predictive control for integrated control of air-conditioning and mechanical ventilation, lighting and shading systems. Applied Energy.

[9] Buffa, S., Fouladfar, M. H., Franchini, G., Lozano Gabarre, I. & Andrés Chicote, M. Advanced control and fault detection strategies for district heating and cooling systems–a review. *Applied Sciences* **11**, 10.3390/app11010455 (2021).

[10] Vandermeulen A, van der Heijde B, Helsen L (2018). Controlling district heating and cooling networks to unlock flexibility: A review. Energy.

[11] Jieyang P (2022). Journal of Intelligent Manufacturing.

[12] Schwenzer M, Ay M, Bergs T, Abel D (2021). Review on model predictive control: an engineering perspective. The International Journal of Advanced Manufacturing Technology.

[13] Huang T, Bacher P, Møller JK, D’Ettorre F, Markussen WB (2023). A step towards digital operations–a novel grey-box approach for modelling the heat dynamics of ultra-low temperature freezing chambers. Applied Energy.

[14] IBM. What is a data pipeline? https://www.ibm.com/topics/data-pipeline (2023).

[15] Apache. Apache parquet. https://parquet.apache.org/ (2023).

[16] Huang T, Nøstvik S, Bacher P, Markussen WB, Møller JK (2023). Figshare.

[17] Huang T, Nøstvik S, Bacher P, Markussen WB, Møller JK (2023). GitLab.

[18] Tan H, Xu L, Yang L, Bai M, Liu Z (2022). Environmental Science and Pollution Research.

[19] Navarro E, Corberán J, Martínez-Galvan I, Gonzálvez J (2012). Oil sump temperature in hermetic compressors for heat pump applications. International Journal of Refrigeration.

[20] Fan C, Xiao F, Li Z, Wang J (2018). Unsupervised data analytics in mining big building operational data for energy efficiency enhancement: A review. Energy and Buildings.

[21] Palmer Real J (2021). Characterisation of thermal energy dynamics of residential buildings with scarce data. Energy and Buildings.

[22] Rouchier S, Rabouille M, Oberlé P (2018). Calibration of simplified building energy models for parameter estimation and forecasting: Stochastic versus deterministic modelling. Building and Environment.

